# Firm-Level Perspectives on Public Sector Engagement with Private Healthcare Providers: Survey Evidence from Ghana and Kenya

**DOI:** 10.1371/journal.pone.0027194

**Published:** 2011-11-23

**Authors:** Neeraj Sood, Nicholas Burger, Joanne Yoong, Dan Kopf, Connor Spreng

**Affiliations:** 1 University of Southern California, Los Angeles, California, United States of America; 2 RAND Corporation, Arlington, Virginia, United States of America; 3 World Bank, Washington, D.C., United States of America; Kenya Medical Research Institute - Wellcome Trust Research Programme, Kenya

## Abstract

**Background:**

Health systems in Sub-Saharan Africa (SSA) are in urgent need of improvement. The private health sector is a major provider of care in the region and it will remain a significant actor in the future. Any efforts by SSA governments to improve health systems performance therefore has to account for the private health sector. Regional and international actors increasingly recognize importance of effectively engaging with the private health sector, and initiatives to improve engagement are underway in several countries. However, there is little systematic analysis of private health providers' view and experience with engagement.

**Methodology/Principal Findings:**

In this study we surveyed private health facilities in Kenya and Ghana to understand the extent to which and how governments interact and engage with these facilities. The results suggest that government engagement with private health facilities is quite limited. The primary focus of this engagement is “command-and-control” type regulations to improve the quality of care. There is little attention paid to building the capacity of health care businesses through either technical or financial assistance. The vast majority of these facilities also receive no government assistance in meeting public health and social goals. Finally, government engagement with private pharmacies is often neglected and clinics receive a disproportionate share of government assistance.

**Conclusions/Significance:**

Overall, our findings suggest that there may be considerable untapped potential for greater engagement with private health facilities—particularly pharmacies. Improving engagement will likely help governments with limited resources to better take advantage of the private sector capacity to meet access and equity objectives and to accelerate the achievement of the Millennium Development Goals.

## Introduction

Across the developing world, the private sector has a well-documented presence in healthcare. In sub-Saharan Africa private providers account for as much as 50 percent of health care provision [1]. Yet the appropriate role for the private health sector remains a controversial issue. While users may prefer the private sector due to perceived quality, easier access and greater responsiveness, in some cases technical standards of care might be poor [2]–[6] and private sector provision may limit access for the poor [7], [8]. Others argue that budget-constrained national health systems may be best served by making the most of the private health sector [9], [10] [11]. Recent evidence from 34 sub Saharan countries also suggests that increased private sector participation may be associated with greater access to services as well as greater equity [12].

Although the debate about the role of the private sector in health care is far from resolved, governments, international organizations and donors have begun to work more with the private health sector since the 1980s [7]. For developing country policymakers, given the size and contributions of the private health sector, a policy of engagement might no longer be just an option but a necessary step towards achieving large-scale improvements in public health. The central question is therefore not whether to interact with the private health sector, but how to best do so in a manner that produces desirable outcomes. Lagomarsino et al. (2009) [13] argue that it is important for policymakers to remain focused on effective public stewardship—i.e. setting and enforcing the rules and incentives that define the environment and guide the behaviors of health-system players, an issue on which there is relatively strong consensus and hence opportunity for progress. However, rigorous evaluations of different approaches to engage with the private sector remain sparse, and are confined largely to case studies [7], [14] [15].

In spite of the perception that many governments fail to constructively engage the private sector due to political, administrative, and information constraints, systematic evidence on the many facets of government-provider interaction remains limited [13]. Much of the literature focuses primarily on the experience of government agencies and consumers, and none has clearly documented government engagement from the perspective of providers.

In this paper, we provide new evidence about government engagement with private sector clinics and pharmacies in two countries, Ghana and Kenya, through the lens of survey data collected from these providers. Our sample of pharmacies and clinics captures the most prevalent providers of health services, though not the full variety of providers. For example, across the region at least one third of health services are provided by informal providers [13], which are largely absent in our sample. To structure the discussion, we first provide a general framework for conceptualizing government engagement. Next, we describe the context for our study and the providers included in the study. We then present the main findings of the paper and draw a number of conclusions about the specific setting of our study as well as more general implications and recommendations for research going forward.

### Public-Private Engagement: Influencing Firms

Prior studies have identified a number of key strategic tools that governments frequently employ under the scope of public-private engagement [16], [17]. These can be thought of as falling into three categories: demand-side interactions that aim to influence consumer behavior (such as social marketing), supply-side interactions influencing firm behavior (such as direct purchase or training), and restructuring or reorganizing market interactions (such as the creating new public-private entities).

This paper characterizes supply-side interactions that are intended to influence firm behavior in two primary domains: (1) increasing overall access and equity in access to health care, and (2) increasing the quality of health care services. We distinguish between the following approaches, which in practice may vary across domains as well as provider types. As an aside, We acknowledge that interventions and supply-side interactions in particular can be categorized differently. The breakdown offered here provides a chance to highlight the aspects our research focused on, while being largely compatible with other standard frameworks [18].

#### Monitoring: Gathering information on the activities of the private sector

At the most fundamental level, governments seeking to engage with the private sector need to know the size and composition of the private health sector. They also need to have the ability to monitor its activity on an ongoing basis. Examples of this include administrative reporting on the number of providers and types/volume of patients they treat and routinely collecting and assessing information on adverse events or patient complaints. This is a crucial aspect of engagement for three reasons. First, such information is crucial for planning and implementing public health programs, especially in countries where private providers are an important access point for health care delivery. For example, it is difficult to establish effective disease surveillance programs without knowing the disease burden for patients being treated in private health facilities. Second, information on private sector size and composition is key for understanding how public policy and other macroeconomic factors are shaping the behavior of the private sector. Finally, without such information it is nearly impossible to evaluate the intended and unintended consequences of other forms of engagement with the private health sector.

#### Information Provision/Technical Assistance: Supplying information to support the activities of the private sector

Firms may fail to maximize their potential scope of business or to comply with appropriate quality standards if they lack the requisite information or training. Simply providing information to the private sector can be a straightforward but important aspect of capacity building and can improve both access to care and quality of care. For example, lack of knowledge about formal credit markets and financial management can be a significant barrier to operating or expanding a health care facility. Government technical assistance programs can reduce such barriers by providing the necessary skills. Ultimately, improved facility performance and growth—induced by government technical assistance—will increase access to care. Similarly, governments can provide technical assistance to promote quality of care by disseminating information on standards of care or best practice guidelines and by providing continuing education workshops or classes that are open to both the public and private sector.

These investments in improving the skills of private health providers are relatively uncontroversial in that the role of the government may be interpreted as simply increasing the flow or supply of information.

#### Financial Assistance: Subsidies, contracting and direct purchase

A more interventionist approach is providing direct financial assistance to the private health sector in the form of grants, subsidies or government contracts to purchase health care services. Examples of financial assistance include land grants for improving access or subsidies for purchasing or upgrading medical equipment to improve quality. Waters et al. (2003) [17] and Loevinsohn and Harding (2003) [19] note a number of examples of contracting-out for primary care and other child health services, from Senegal, Madagascar, El Salvador, Cambodia, Guatemala, South Africa and Zimbabwe. While a majority of these experiences involve NGOs, a growing number also involve for-profit contractors. Sometimes financial assistance can be “performance based” in that it is tied to achievement of specific outcomes or provision of specific services. For example, private providers who report providing care to a target number of rural patients may receive a supplementary allowance or priority in receiving loans. Governments may also provide incentives to private facilities for the provision of public health goods such as health education, vaccination and other interventions to prevent infectious diseases.

#### Regulation

Government can choose to implement “command-and-control” style rules and penalties and thereby exercise its authority to require and enforce what it considers to be desirable practice. Regulation can apply to measures to protect the quality of care, for instance by establishing minimum cleanliness standards and penalties for violations. It can also be used to expand access and increase equity, for example by imposing price controls or requirements that private providers exempt certain types of patients from fees. Regulatory approaches are limited, however, in that they often treat all regulated entities the same and may not be economically efficient.

While potentially more powerful than monitoring and information provision, financial assistance and regulation can have unintended consequences. For example, price controls to improve access might deter provider entry into health care markets and thus reduce access in the long run. Prior studies of price controls in China document numerous other unintended consequences including the emergence of a black market, overprovision of profitable high-tech services, and overuse of prescription drugs [20]. Similarly, subsidies and financial assistance to providers might promote inefficiency by using public funds to bolster inefficient providers. As with most public programs, such interventions may be prone to error, fraud and corruption, as well as susceptible to capture by special interest groups. Using performance based incentives can mitigate some of these concerns but may not offset others: for instance, targets may be inappropriately set, or measurement and compliance may be manipulated for gain.

Given the tradeoffs across different types of engagement policies, the debate over the extent and nature of government intervention is domain and context specific. For instance, sensitivity to the issues above: may be greater when policies are aimed at providing financial incentives to private facilities to promote access rather than policies aimed at upholding quality standards; may depend on the type of provider; or may be linked to overall governance in the country. It is important to note that the approaches described above may be initiated or directed by entities that are not part of the government. Private health sector associations and third parties (e.g., accreditation agencies, insurance agencies) may engage in some or all of the approaches. In this paper, however, we leave the role of associations aside. The goal of this paper is to characterize the approaches taken by the governments of Ghana and Kenya, as reportedly experienced by private sector providers.

## Methods

### Study sample and location

The nature of health systems and the environment in which patients seek care and firms do business varies tremendously from country to country, across the developing world. Within any given country the private health sector encompasses a diverse set of providers, divided by many distinctions, including position on the supply-chain, for-profit status, religious/secular affiliation, degree of formality, and participation in allopathic or traditional medical practice [17], [21]. Any discussion of engagement can be specific to a particular subset of this group.

### Sample description

The data used for the study come from the Health Provider Assessment Survey, which was administered in Ghana and Kenya during 2010 by the study team. HPAS samples for each country were designed to capture a broad range of health facility types, focusing primarily on smaller, private sector firms. In Ghana, the sampling frame was based on a 2010 census of health facilities in seven districts purposively chosen to be geographically and economically diverse, carried out by the Results for Development Institute. We excluded laboratories and medical device manufacturers and out of the remaining 647 facilities, we interviewed a random sample of 300 hospitals, clinics, nursing homes and pharmacies. Private hospitals and clinics were oversampled. In Kenya, we constructed a census of health facilities in five districts also reflective of geographic and economic diversity, by combining a list of 1920 hospitals, clinics, and nursing homes compiled by the Ministry of Health and KEMRI-Wellcome Trust with a list of 1948 pharmacies from a retail census collected by TNS Opinion. Similarly, we interviewed a random sample of 300 hospitals, clinics, nursing homes and pharmacies drawn from this census, oversampling private hospitals and clinics.

Table 1 shows the final HPAS survey composition by provider type in each country. We note that response rates for the survey differed across countries - 90 percent in Ghana and 69 percent in Kenya - but we do not have any evidence of differential self-selection affecting the final sample composition.

**Table 1:** HPAS sample composition by country.

	Kenya		Ghana	
	Public	Private	Public	Private
Hospital	1	10	8	21
Clinic	11	112	31	68
Pharmacy	1	145	0	92
Chemical Sellers	0	6	0	80
Nursing/maternity homes	0	7	0	0
Other	5	2	0	0
	18	282	39	261

In this paper, we focus on private clinics and pharmacies. We note that because chemical sellers are not a separate class of providers for regulatory purposes in Kenya (but not in Ghana), we include the small number of Kenyan providers that identify as such under the category of pharmacies. In Ghana, the analytical sample therefore consisted of 68 clinics and 92 pharmacies. In Kenya, the sample consisted of 112 clinics and 151 pharmacies.

### HPAS Survey Questions

HPAS survey questions are grouped into five core sections: basic facility characteristics, barriers and obstacles to operating a business, the policy environment, financial information, and business process management. In Ghana we included a supplemental section regarding the national health insurance scheme, and in Kenya the survey contained a supplemental section specific to pharmacies. A final section asks enumerators to provide a basic assessment of the facility, including information on amenities and cleanliness.

With respect to interaction with the government, the HPAS instrument asks questions to providers about various aspects of regulation and about their experience with government assistance to build their human and financial capital. The survey also asks providers whether they have been exposed to financial incentive schemes and about the nature of partnerships with the government on certain public health activities.

#### Monitoring

The HPAS asks providers whether they report information about their activities to the government across a variety of domains related to health management systems reporting, epidemiological surveillance-oriented reporting, and business operations. In particular, the survey asks providers whether they send periodic reports to the government on each of the following: (1) service and drug utilization statistics on the number or types of patients seen and the number or types of drugs sold, (2) adverse events such as maternal or child deaths, (3) quality reporting on compliance with standards, and (4) financial reporting on operating information such as prices and revenues.

#### Technical and Financial Assistance for Capacity Building

Providers in both countries are also asked a series of questions about supportive resources received from the government, whether in the form of information, technical assistance or financial assistance. In particular, the survey asks providers about assistance (technical or financial) to support them in two types of activities, during the past three years:

#### Skill and Technology Upgrades

Providers are asked about offers of assistance (technical or financial) to support them in five domains: (1) continuing education for existing providers, (2) training for future health providers, (3) information about clinical practice guidelines, (4) quality assurance practices, and (5) technology upgrades. A follow-up question for those offered assistance verified whether or not such assistance was actually received.

#### Raising Capital

Providers are asked about whether they receive any technical assistance from the government to support them in improving their ability to apply for a bank loan, or whether they received direct financial assistance such as a loan, grant, subsidized interest, or bank guarantee.

#### Regulation

In the case of regulation and its enforcement, the HPAS asks questions that cover two areas: access/equity and quality.

#### Access/equity

Providers are asked if their facility is subject to laws and regulations that mandate (1) the need to provide free or subsidized care to poor patients, (2) inability to deny treatment based on cost/ability to pay, and (3) price caps or maximum price regulations that mandate that prices cannot exceed a certain threshold.

#### Quality

Providers are asked about whether they have had an inspection from the government in the past two years for monitoring the safety and quality of their services. With respect to enforcement, since facilities may be reluctant and unlikely to answer truthfully questions about failing inspections, they are also asked an indirect question about whether they know of any examples of facilities like theirs that have been penalized by the government for failing to meet quality standards (with examples given such as selling counterfeit drugs or causing adverse health events).

#### Technical and Financial Assistance for Improving Public Health and Reducing Disparities

As an alternative to mandates providers are also asked about government-provided incentives to achieve public health and broader social objectives.

#### Access to Public Health Services

Providers are asked about whether in the past 3 years they have received any technical or financial assistance from the government to provide services related to public health, specifically: (1) childhood vaccinations, (2) HIV/AIDS control measures, (3) malaria or tuberculosis (TB) control measures, and (4) health education for consumers/patients.

#### Equity

Providers are asked about whether they know of any facilities that receive help from the government for (1) opening a facility in a rural area, (2) opening a facility in a poor urban area, and (3) providing treatment to poor patients.

## Results

### Monitoring

**Figure 1:**
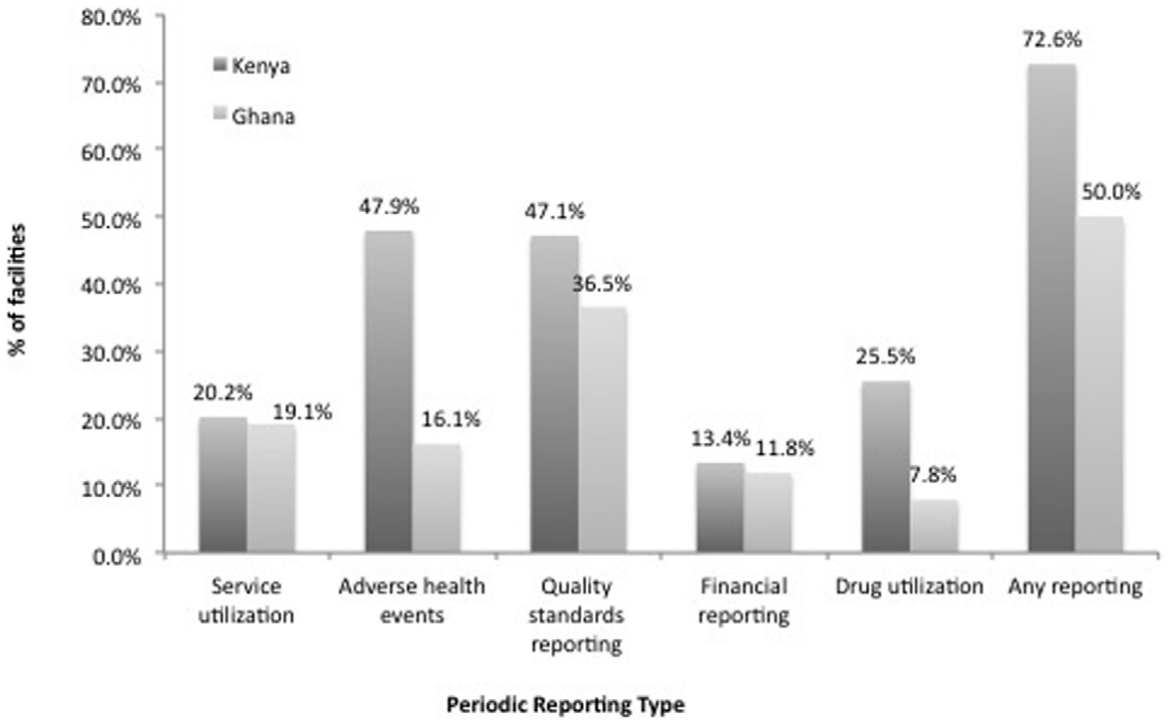
Periodic Reporting Type. Countries: • Dark Grey: Kenya • Light Grey: Ghana.

Figure 1 below shows responses to the questions about monitoring in both countries, with the Kenyan sample showing slightly higher rates of overall contact. In Kenya, almost 30% and in Ghana half of the facilities in the sample stated that they participated in *none* of the reporting activities described in the previous section—although results are not consistent across all categories. Interestingly, in both countries, facilities are most likely to report information to the government about quality standards and somewhat less likely to report on utilization, both in terms of patient and drug turnover. Finally, an even smaller number of facilities report any financial information to the government −13% in Kenya and 12% in Ghana. These findings suggest that the limited monitoring that does exist focuses on the medical aspects of these facilities but largely neglects their operation as businesses.

In Table 2, these figures are broken down by facility type in each country. In both countries clinics are more likely than pharmacies to report their activities to the government. One exception is reports related to drug inventory activity in Kenya, where pharmacies report higher levels of reporting. In Ghana, pharmacies are only marginally more likely to report drug inventory relative to clinics. Few facilities—of either type and in either country—report financial information to the government.

**Table 2:** Facilities providing periodic reports to the government, by type.

	Kenya				Ghana			
	Clinics		Pharmacies		Clinics		Pharmacies	
	*N*	%	*N*	%	*N*	%	*N*	%
Service utilization (number and type of patients)	*112*	35.7%	*151*	8.6%	*67*	40.3%	*90*	3.3%
Epidemiological reporting: adverse events	*112*	59.8%	*151*	39.1%	*66*	24.2%	*89*	10.1%
Quality standards reporting	*112*	54.5%	*151*	41.7%	*67*	43.3%	*89*	31.5%
Financial reporting	*112*	13.4%	*150*	13.3%	*66*	10.6%	*86*	12.8%
Drug utilization (number and type of drugs)	*112*	19.6%	*151*	29.8%	*66*	7.6%	*87*	8.0%
*Any reporting*	*112*	75.9%	*151*	70.2%	*67*	59.7%	*91*	42.9%

Note: “Don't know” and refusals are both treated as missing.

In summary, the level of government monitoring of private health facilities across a variety of domains is poor. Clinics are more likely to be monitored than pharmacies. Domains related to quality of care are more likely to be monitored than others, but even for these domains the majority of facilities are not monitored.

### Technical Assistance for Capacity Building

#### Skill and Technology Upgrades

**Figure 2:**
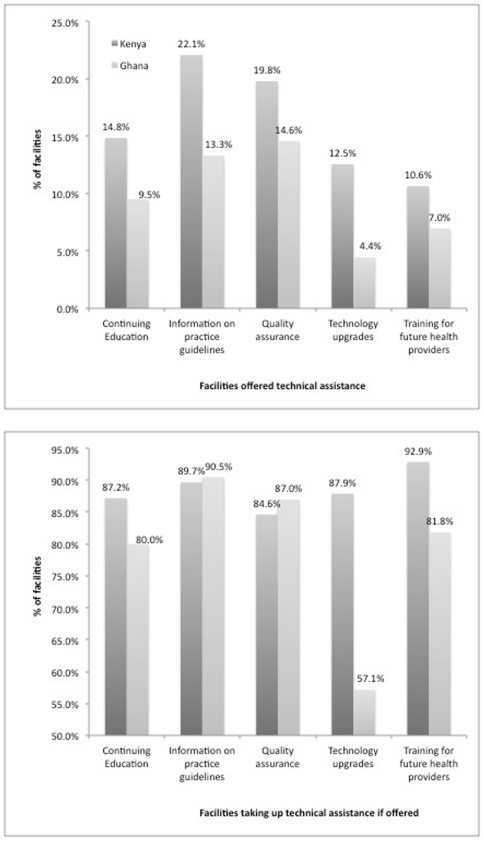
(top): Facilities Offered Technical Assistance. (bottom): Facilities Taking Up Technical Assistance if Offered. Countries: • Dark Grey: Kenya • Light Grey: Ghana.

We first examine government interaction with private facilities regarding technical assistance related to improving provider skills and technology. The results can be found in Figure 2 below.

From the top panel of Figure 2, we can see that surprisingly few facilities receive offers of support related to upgrading human capital whether in the form of continuing education or training of future providers (between 7–15% in either case for both countries). Similarly, less than 15% of facilities in either country report receiving offers of technology upgrades. Relatively more facilities are offered assistance related to maintaining quality and practice standards, although in each case this still reflects less than a quarter of all facilities.

The bottom panel of Figure 2 shows that the majority of facilities participate and take advantage of these services if offered. For facilities not taking up these services, it is not possible from the data to understand whether it is the result of refusing offered assistance, or whether it is due to the government's failure to follow up.

Table 3 shows an important divergence in these results by facility type. In both Ghana and Kenya, the offers of technical support across all dimensions are significantly skewed towards clinics; yet the take up of technical assistance among clinics and pharmacies is more comparable.

**Table 3:** Facilities offered/receiving technical assistance for skill and technology upgrades from the government in last 3 years, by type.

	Kenya				Ghana			
	Clinics		Pharmacies		Clinics		Pharmacies	
	N	%	N	%	N	%	N	%
*Facilities being offered technical support for*								
Continuing Education	*112*	25.0%	*151*	7.3%	*68*	13.2%	*90*	6.7%
Information on practice guidelines	*112*	40.2%	*151*	8.6%	*68*	20.6%	*90*	7.8%
Quality assurance	*112*	32.1%	*151*	10.6%	*68*	22.1%	*90*	8.9%
Technology upgrades	*112*	23.2%	*151*	4.6%	*68*	8.8%	*90*	1.1%
Training for future health providers	*112*	16.1%	*151*	6.6%	*68*	8.8%	*90*	5.6%
*Facilities receiving technical support (as % offered)*								
Continuing Education	*28*	85.7%	*11*	90.9%	*9*	77.8%	*6*	83.3%
Information on practice guidelines	*45*	91.1%	*13*	84.6%	*14*	85.7%	*7*	100.0%
Quality assurance	*36*	83.3%	*16*	87.5%	*15*	80.0%	*8*	100.0%
Technology upgrades	*26*	88.5%	*7*	85.7%	*6*	50.0%	*1*	100.0%
Training for future health providers	*18*	94.4%	*10*	90.0%	*6*	66.7%	*5*	100.0%

Note: “Don't know” and refusals are both coded as missing values.

#### Raising Capital

Virtually no facilities report receiving technical assistance to improve their ability to apply for bank loans. Specifically, 3.8% of facilities in Kenya and no facilities in Ghana report receiving such technical assistance (not reported in tables).

In summary, the provision of technical assistance by the government to private health facilities is fairly uncommon with more than three quarters of these facilities reporting not receiving technical assistance. The provision of technical assistance for improving access to private credit markets is virtually non-existent. Clinics are more likely to receive technical assistance compared to pharmacies.

### Financial Assistance for Capacity Building

**Figure 3:**
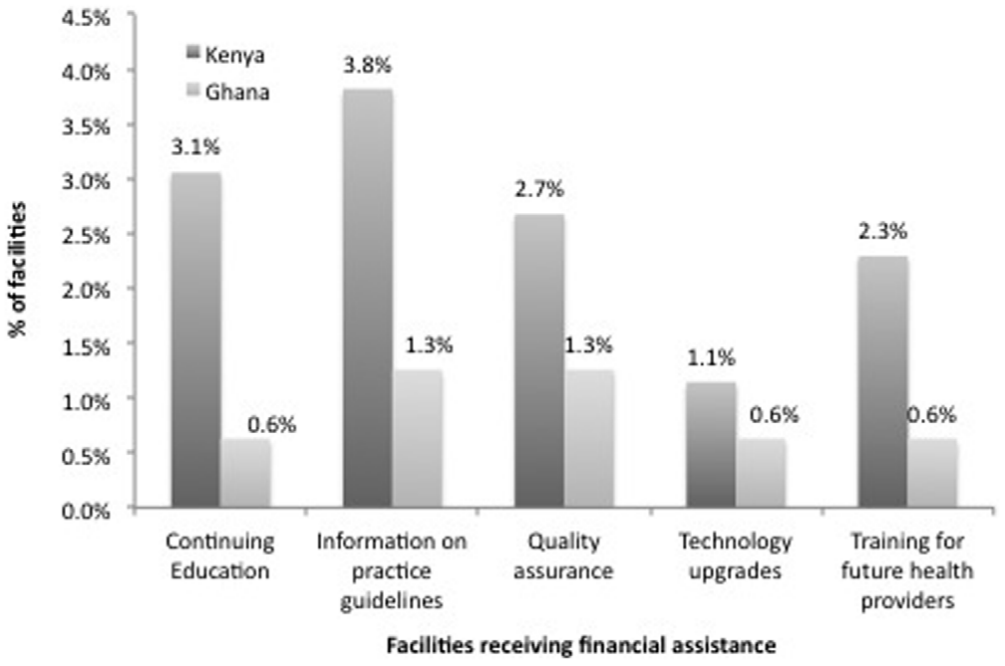
Facilities Receiving Financial Assistance. Countries: • Dark Grey: Kenya • Light Grey: Ghana.

Figure 3 shows that in both countries only a small minority of facilities (5% or fewer) report receiving direct financial support from the government for improving provider skills and technology upgrades. However, across all domains, financial support is more widely reported in Kenya than Ghana.

Again, in Table 4, we observe that, as with technical assistance, what little financial assistance exists is skewed towards clinics rather than pharmacies. Virtually no pharmacies in Kenya and no pharmacies at all in Ghana report receiving financial assistance for continuing education, training or technology upgrades.

**Table 4:** Facilities receiving financial assistance for operating activities from the government in last 3 years, by type.

	Kenya				Ghana			
	Clinics		Pharmacies		Clinics		Pharmacies	
	N	%	N	%	N	%	N	%
*Facilities receiving financial assistance for*								
Continuing Education	*111*	6.3%	*151*	0.7%	*67*	1.5%	*92*	0.0%
Information on practice guidelines	*111*	6.3%	*151*	2.0%	*67*	3.0%	*92*	0.0%
Quality assurance	*111*	6.3%	*151*	2.0%	*67*	3.0%	*92*	0.0%
Technology upgrades	*111*	1.8%	*151*	0.7%	*67*	1.5%	*92*	0.0%
Training for future health providers	*111*	3.6%	*151*	1.3%	*67*	1.5%	*92*	0.0%

Note: “Don't know” and refusals are both coded as missing values.

### Regulation

#### Access/Equity

**Figure 4:**
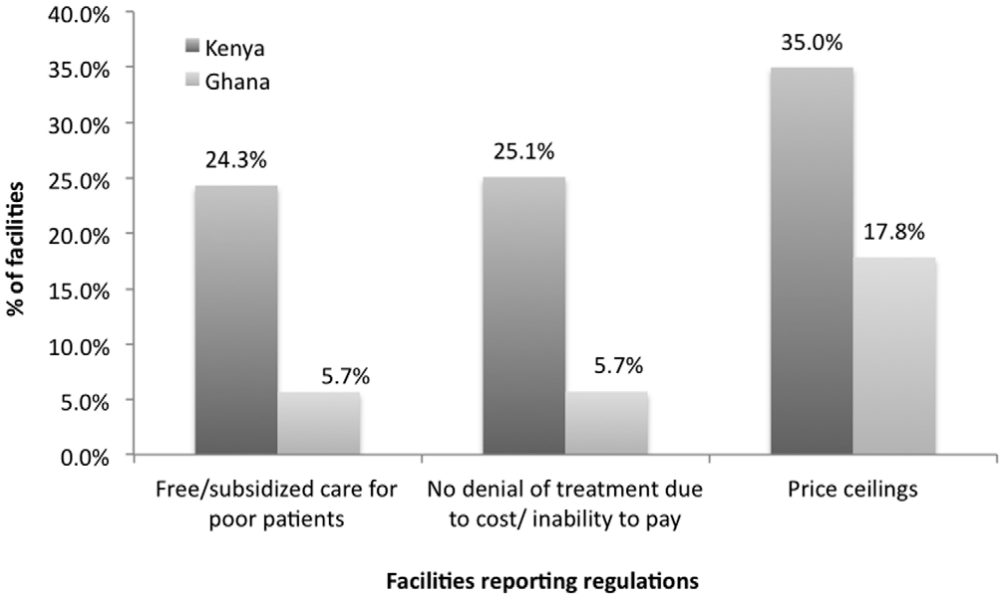
Facilities Reporting Regulations. Countries: • Dark Grey: Kenya • Light Grey: Ghana.

In both Ghana and Kenya, regulation with respect to equity-enhancing mandates is reported only by a minority of facilities (Figure 4). The results clearly point to the existence of an overall regulatory structure. However, in Kenya only a quarter to a third of all facilities report being subject to such mandates. In Ghana the percentages are even lower. Overall, though the levels differ, price controls are most commonly reported, while mandates related to the provision of care based on poverty and the inability to refuse care are much less frequently reported in both countries (Figure 4). Table 5 shows that in Kenya, a comparable if not equal amount of regulation appears to apply to clinics and pharmacies. This is in sharp contrast to Ghana, where 19% of pharmacies report being to be subject to price controls but otherwise the sector seems to be relatively untouched by regulations mandating free or subsidized care.

**Table 5:** Facilities reporting being subject to access/equity-related regulation, by type.

	Kenya				Ghana			
	Clinics		Pharmacies		Clinics		Pharmacies	
	N	%	N	%	N	%	N	%
*Facilities reportedly subject to regulation mandating*								
Free/subsidized care for poor patients	*112*	24.1%	*151*	24.5%	*67*	11.9%	*92*	1.1%
No denial of treatment due to cost/inability to pay	*112*	28.6%	*151*	22.5%	*67*	10.4%	*91*	2.2%
Price ceilings	*112*	31.3%	*151*	37.7%	*67*	16.4%	*90*	18.9%

Note: “Don't know” and refusals are both coded as missing values.

#### Quality regulation

**Figure 5:**
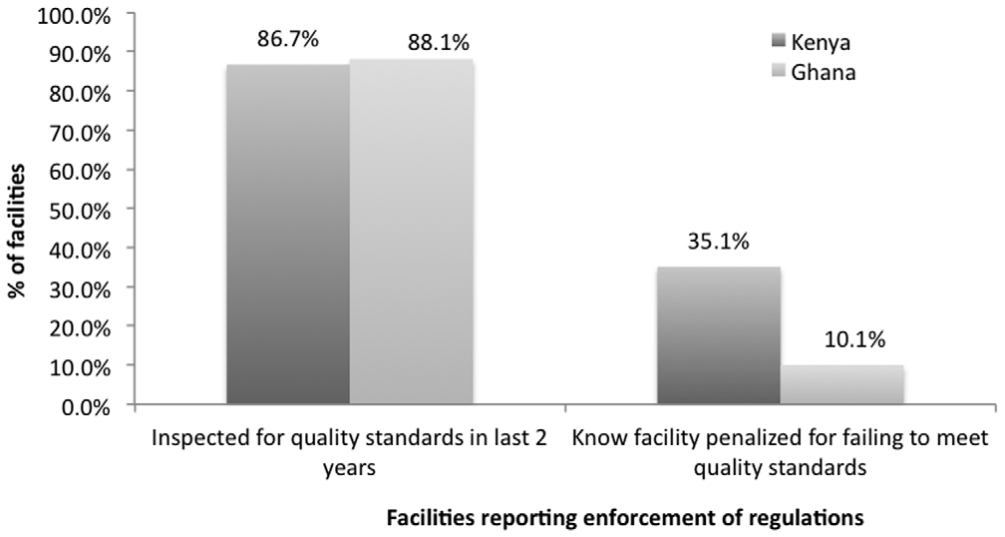
Facilities Reporting Enforcement of Regulations. Countries: • Dark Grey: Kenya • Light Grey: Ghana.

Almost 90% of firms in both countries report having been inspected in the last 2 years. 35% of Kenyan facilities but only 10% of Ghanaian facilities report having heard of a case in which a facility like theirs has been penalized for not meeting quality standards (Figure 5). The breakdown by facility type (Table 6) shows that in both countries, clinics and pharmacies report inspection rates of almost 80% or more. In Ghana, pharmacies are both more likely to be inspected, and more likely to have heard about penalties imposed. However, in Kenya, pharmacies are about 10% less likely to be inspected relative to clinics, but more than twice as likely to have heard of someone being penalized.

**Table 6:** Facilities reporting enforcement of quality-related regulation, by type.

	Clinics		Pharmacies		Clinics		Pharmacies	
	N	%	N	%	N	%	N	%
Inspected for quality standards in last 2 years	*112*	91.1%	*151*	83.4%	*68*	79.4%	*92*	94.6%
Know facility penalized for failing to meet quality standards	*111*	19.8%	*151*	46.4%	*68*	5.9%	*91*	13.2%

Note: “Don't know” and refusals are both coded as missing values.

### Technical and Financial Assistance for Improving Public Health and Reducing Disparities

#### Supporting Access to Public Health Services

**Figure 6:**
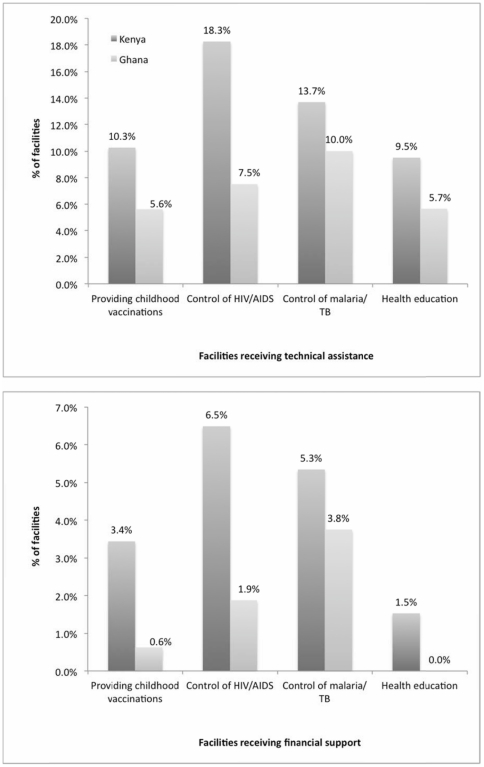
(top): Facilities Receiving Technical Assistance. (bottom): Facilities Receiving Financial Support. Countries: • Dark Grey: Kenya • Light Grey: Ghana.

A visible but small minority of private sector providers are incentivized by the government in both countries to support them in providing health services that are public goods (Figure 6); the percentage of facilities involved however is no more than 10–20% for any type of service covered by the survey in either country.

Comparing the top and bottom panel of Figure 6 shows that in terms of incentivizing service delivery, technical assistance is more widespread than financial assistance (similar to the case of capacity building). Overall, in both countries, the smallest number of facilities report being assisted in the provision of general health education, but interestingly, this fraction is not much smaller than the other categories. (We note, however, that our measure does not capture the magnitude of funding, and we thus miss an important dimension of the intensity with which the private sector is supported). Table 7 shows that clinics are more likely than pharmacies to receive services in this domain as well.

**Table 7:** Facilities receiving technical/financial assistance from the government to provide public health services in last 3 years, by type.

	Kenya				Ghana			
	Clinics		Pharmacies		Clinics		Pharmacies	
	N	%	N	%	N	%	N	%
*Facilities receiving technical assistance for*								
Providing childhood vaccinations	*112*	18.8%	*151*	4.0%	*68*	11.8%	*92*	1.1%
Control of HIV/AIDS	*112*	34.8%	*151*	6.0%	*68*	14.7%	*92*	2.2%
Control of malaria/TB	*112*	25.9%	*151*	4.6%	*68*	16.2%	*92*	5.4%
Health education	*112*	17.0%	*151*	4.0%	*68*	10.3%	*91*	2.2%
*Facilities receiving financial support for*								
Providing childhood vaccinations	*111*	8.1%	*151*	0.0%	*68*	1.5%	*92*	0.0%
Control of HIV/AIDS	*111*	13.5%	*151*	1.3%	*68*	4.4%	*92*	0.0%
Control of malaria/TB	*111*	11.7%	*151*	0.7%	*68*	4.4%	*92*	3.3%
Health education	*111*	3.6%	*151*	0.0%	*68*	0.0%	*92*	0.0%

Note: “Don't know” and refusals are both coded as missing values.

Overall, it appears that there is a large fraction of the private sector that could potentially be further incentivized to help achieve public health goals; this is particularly salient given that three of the four measures above contribute directly to the achievement of the Millennium Development Goals.

#### Equity

**Figure 7:**
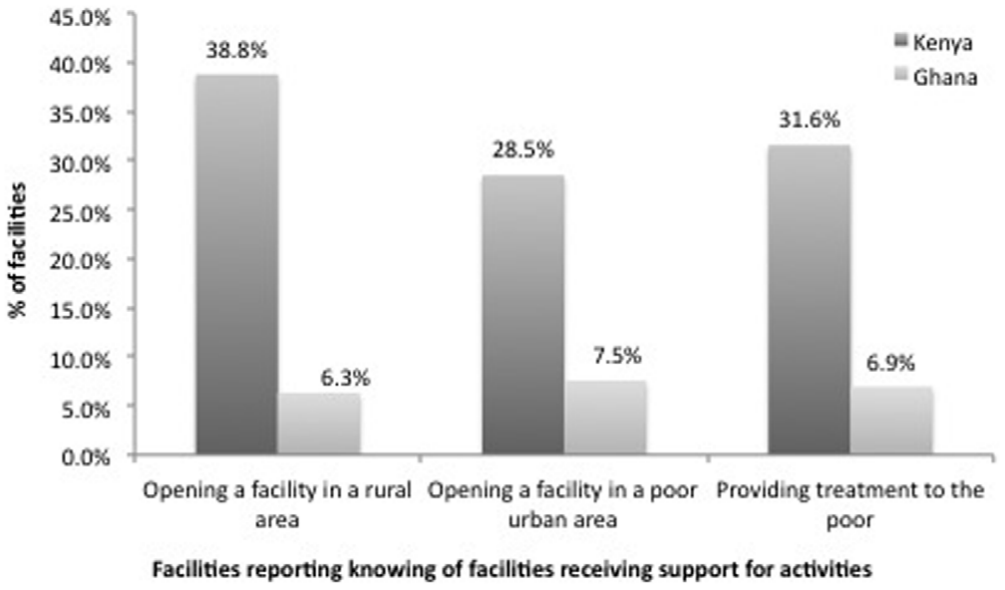
Facilities Reporting Knowing of Facilities Receiving Support for Activities. Countries: • Dark Grey: Kenya • Light Grey: Ghana.

Figure 7 shows the percentage of facilities reporting that they have heard of another facility receiving financial support to promote service provision activities that address equity issues. Rates are considerably higher in Kenya rather than Ghana, particularly for promoting service in rural areas. In Kenya, nearly 40% of facilities reported knowing a facility that has received assistance to serve a rural area. About 30% reported knowing a facility that has received assistance to serve a poor urban area and a slightly higher number reported knowing a facility that has received assistance for treating the poor. In Ghana, these numbers are significantly lower, at 6–7% for all three categories.

Table 8 shows that the difference between the two counties manifests not only as higher average response rates in Kenya overall, but also in a markedly different environment in the pharmacy sector. In Ghana, consistent with the previous results, pharmacies appear to be much less engaged than clinics. However, in Kenya, pharmacies appear to be more frequently incentivized (or have better knowledge of incentives being provided) relative to clinics in the same country, and relative to pharmacies in Ghana, by a wide margin −45% report knowing a facility that has received assistance to serve a rural area, 36% reported knowing a facility that has received assistance to serve a poor urban area and 39% reported knowing a facility that has received assistance, relative to 30%, 18% and 21% for clinics respectively.

**Table 8:** Facilities reporting knowing of another facility receiving support from the government to promote equity, by type.

	Kenya				Ghana			
	Clinics		Pharmacies		Clinics		Pharmacies	
	N	%	N	%	N	%	N	%
*Facilities reporting knowing another facility receiving support for*								
Opening a facility in a rural area	*112*	30.4%	*151*	45.0%	*67*	10.4%	*92*	3.3%
Opening a facility in a poor urban area	*112*	17.9%	*151*	36.4%	*67*	11.9%	*92*	4.3%
Providing treatment to the poor	*112*	21.4%	*151*	39.1%	*67*	10.4%	*92*	4.3%

Note: “Don't know” and refusals are both coded as missing values.

## Discussion

In this study we surveyed private health facilities in Kenya and Ghana to understand the extent to which and how governments interact and engage with these facilities. The main conclusion from this research is that government engagement with the private health facilities is quite limited. The primary focus of this engagement is command-and-control type regulations to improve the quality of care. There is little attention paid to capacity building for health care businesses through either technical or financial assistance. The vast majority of facilities also receive no government assistance in meeting public health and social goals. Finally, government engagement with private pharmacies is often neglected and clinics receive a disproportionate share of government assistance. For the private health sector to contribute more effectively to national health goals, increasing engagement with private facilities as both businesses and medical care providers will be critical.

A first order problem in both Kenya and Ghana is that the government does little to monitor the activity of the private sector in terms of the medical care provided and the finances of these facilities. Such a widespread lack of information about the private sector implies that governments in these countries have little ability to assess the effectiveness of public policies targeted towards the private health sector. The lack of monitoring of private health sector activity also suggests that the governments have an incomplete and dated view of the role the private sector is playing in these economies.

The nature of the interactions most commonly-reported in the survey suggests that, from the firm's perspective, most government intervention in these countries still takes a cautious approach, viewing the private sector as a liability rather than an opportunity. Policies tend to focus more strongly on controlling the quality rather than fostering the private sector to meet access goals. Across the board, direct technical assistance is more prevalent across all types of capacity building than financial sponsorship of such activities, possibly due to logistical feasibility or ease of control. Many firms report receiving incentives to promote quality or being subject to regulation and inspection to ensure quality, but fewer report being involved with interventions to support expansion, even if this promotes access and equity outcomes.

Finally, when contrasting engagement across provider types, it appears that across all types of interventions, in both countries, pharmacies are relatively excluded. In both Ghana and Kenya, government support for capacity building seems largely confined to clinics. Pharmacies reported virtually no financial support, and significantly less technical assistance, with some notable exceptions such as incentives to expand into underserved areas in Kenya. The relative lack of interaction with small retail pharmacies points to a need for policy reform in this area, given the large fraction of consumers, especially the poor, that report receiving health services primarily or even exclusively from pharmacies.

Some important limitations should be noted, as well as their implications for the findings reported here and for further research. The firms in our sample were largely formally registered, implying that the channels for communication between public and private sectors are at least in theory open and available. The survey did not investigate engagement with many informal providers. Insofar as levels of government interaction with informal providers are likely to be lower, our results may be interpreted as an upper-bound on engagement activity with the private sector as a whole. This study is descriptive, but further research with this dataset will allow us to understand variation in engagement within provider types—e.g., by experience, size—and the degree to which engagement is associated with outcomes for providers.

Overall, our findings suggest that there may be considerable untapped potential for greater engagement with private health facilities, particularly pharmacies. Improving this engagement will likely help governments with limited resources to better take advantage of the capacity of the private sector to meet access and equity objectives, and to accelerate the achievement of the Millennium Development Goals.

At the same time we acknowledge that lack of engagement could be a function of limited resources, either financial or in terms of technical capacity. Just as many governments struggle to provide public health care services, they may also struggle to act as effective monitors and engaged partners with the private sector. The results presented here raise difficult questions about how to allocate scarce resources: should resources be allocated to additional public provision or to better engagement with the private sector? Should additional monitoring resources be focused on small, ubiquitous facilities (i.e., pharmacies) or larger facilities providing more substantial care (i.e., clinics)? These challenges should be addressed by future research.

### Ethics Statement

This study was reviewed and approved by the RAND Human Subjects Protection Committee (HSPC). All survey participants provided verbal informed consent. Verbal consent was approved by the RAND HSPC and required that (1) the interviewer follow an oral script approved by the RAND HSPC when administering the consent process, and (2) the interviewer provide an information letter to survey participants that explains the purpose of the study, the risks and benefits of the study, and provides contact information for the principal investigator of the study and local research team.
